# Influenza-Infected Neutrophils within the Infected Lungs Act as Antigen Presenting Cells for Anti-Viral CD8^+^ T Cells

**DOI:** 10.1371/journal.pone.0046581

**Published:** 2012-10-08

**Authors:** Matthew M. Hufford, Graham Richardson, Haixia Zhou, Balaji Manicassamy, Adolfo García-Sastre, Richard I. Enelow, Thomas J. Braciale

**Affiliations:** 1 The Beirne B. Carter Center for Immunology Research, The University of Virginia, Charlottesville, Virginia, United States of America; 2 Department of Microbiology, The University of Virginia, Charlottesville, Virginia, United States of America; 3 Center for Cell Signaling, The University of Virginia, Charlottesville, Virginia, United States of America; 4 Department of Microbiology, Mount Sinai School of Medicine, New York City, New York, United States of America; 5 Global Health and Emerging Pathogens Institute, Mount Sinai School of Medicine, New York City, New York, United States of America; 6 Department of Medicine, Division of Infectious Diseases, Mount Sinai School of Medicine, New York City, New York, United States of America; 7 Departments of Medicine and Microbiology/Immunology, Dartmouth Medical School, Lebanon, New Hampshire, United States of America; 8 Department of Pathology, The University of Virginia, Charlottesville, Virginia, United States of America; University of Iowa, United States of America

## Abstract

Influenza A virus (IAV) is a leading cause of respiratory tract disease worldwide. Anti-viral CD8^+^ T lymphocytes responding to IAV infection are believed to eliminate virally infected cells by direct cytolysis but may also contribute to pulmonary inflammation and tissue damage via the release of pro-inflammatory mediators following recognition of viral antigen displaying cells. We have previously demonstrated that IAV antigen expressing inflammatory cells of hematopoietic origin within the infected lung interstitium serve as antigen presenting cells (APC) for infiltrating effector CD8^+^ T lymphocytes; however, the spectrum of inflammatory cell types capable of serving as APC was not determined. Here, we demonstrate that viral antigen displaying neutrophils infiltrating the IAV infected lungs are an important cell type capable of acting as APC for effector CD8^+^ T lymphocytes in the infected lungs and that neutrophils expressing viral antigen as a result of direct infection by IAV exhibit the most potent APC activity. Our findings suggest that in addition to their suggested role in induction of the innate immune responses to IAV, virus clearance, and the development of pulmonary injury, neutrophils can serve as APCs to anti-viral effector CD8^+^ T cells within the infected lung interstitium.

## Introduction

Influenza A virus (IAV) is a major cause of severe respiratory viral infections, particularly among the elderly and very young children and can exacerbate pre-existing conditions such as cardiovascular disease [1]. Epithelial cells of the upper and lower respiratory tract are critical for virus propagation as these cells possess a key host protease essential for proper maturation of the viral hemagglutinin receptor and as a result, supports productive infection of the virus. While other cell types (e.g. fibroblasts and cells of hematopoietic origin) can take up IAV virions and support *de novo* viral gene expression [2], [3], in most instances (and for most IAV strains), these infected non-epithelial cell types do not support the production of fully infectious virions. Due to this cellular restriction in viral propagation, IAV-infected respiratory epithelial cells represent critical cellular targets of the host response to IAV infection

Cells of both the innate and adaptive immune system play important roles in the host response IAV infection including the control and elimination of infectious virus and the induction of inflammation and tissue injury associated with virus infection and virus elimination. In the IAV infected murine lungs, effector CD8^+^ T cells primarily control infection and eliminate virally infected cells by direct cytolysis of these infected cells, most notably infected respiratory epithelial cells [4], [5], [6]. These effector CD8^+^ T cells in infected lungs also produce cytokines/chemokines, which may contribute to but are not essential for ultimate virus clearance [7], [8], [9], [10], [11].

Our laboratory has recently demonstrated that in the IAV infected lungs, the cell type recognized by the anti-viral CD8^+^ T cells dictates the spectrum of effector activity [5]. The respiratory epithelium, the cell type supporting productive viral infection, triggered cytolysis by effector CD8^+^ T cells but does not stimulate T cell cytokine production. In contrast, inflammatory cells infiltrating the IAV infected lungs are potent stimulators both of cytolysis and cytokine production by the effector CD8^+^ T cells. We further observed that cytokine production by the effector T cells also required specific IAV antigen recognition and was dependent on expression of co-stimulatory ligands (e.g. CD80 and CD86). We also demonstrated that CD11c^hi^ macrophages and dendritic cells infiltrating the IAV infected lungs were potent APC for cytokine production by effector CD8^+^ T cells. The contribution of any other inflammatory cell type(s) infiltrating the IAV infected lungs as potential APC for effector CD8^+^ T cells was not assessed.

Neutrophils are a prominent component of the inflammatory cell infiltrate accumulating in the IAV infected lungs [12], [13], [14]. Neutrophils have been reported both to facilitate (or indeed even mediate) virus clearance and to modulate pulmonary inflammation and injury associated with the host response to infection [7], [8], [9], [10], [11]. What role, if any, neutrophils play as APC for IAV specific effector CD8^+^ T cells in the infected lungs is at present unknown. Likewise, the susceptibility of neutrophils recruited to the respiratory tract to infection by IAV and the requirement for direct infection of neutrophils for this cell type to serve as an APC in the IAV infected lungs have been largely unexplored to date.

In this report, we evaluated the capacity of immune/inflammatory cells in the IAV infected lungs to take up IAV antigens, the susceptibility of lung infiltrating neutrophils to IAV infection, and the ability of neutrophils to serve as APC for effector CD8^+^ T cells in the infected lungs. We demonstrate that a significant fraction of these lung infiltrating neutrophils are infected by IAV as measured by *de novo* viral gene expression and protein display in these neutrophils. We further demonstrate that viral antigen displaying neutrophils isolated from the IAV infected lungs could act as APC and stimulate cytokine production by effector CD8^+^ T cells isolated from the IAV infected lungs with infected neutrophils exhibiting the most potent APC activity. Consistent with these results, we found that acute *in vivo* depletion of neutrophils significantly reduced soluble mediator production by effector CD8^+^ T cells. Together, these results suggest that neutrophils infiltrating the IAV infected lungs may be potent APC for anti-viral CD8^+^ T cell in the IAV infected lungs.

## Results

### Kinetics of accumulation of innate and adaptive immune cells in the lungs following influenza infection

**Figure 1:**
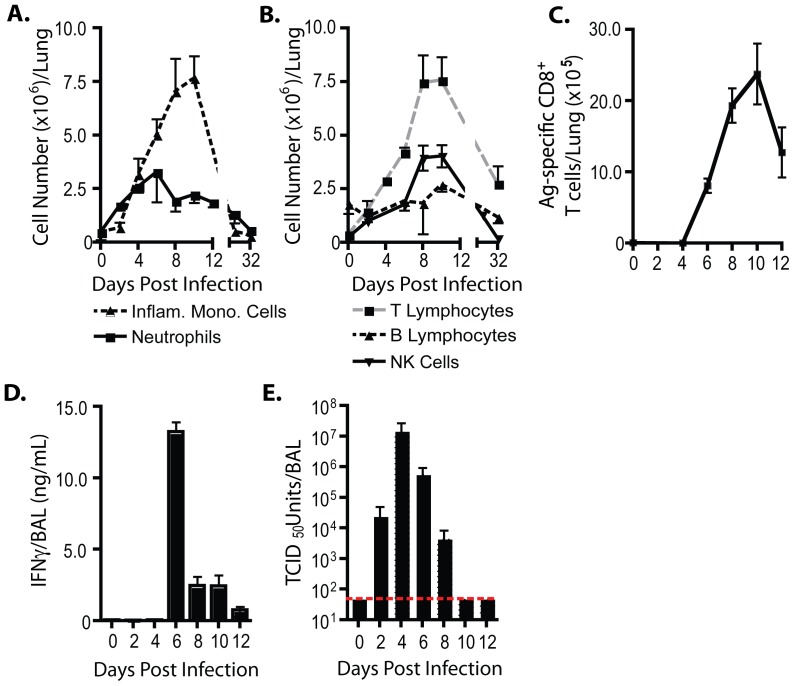
Host immune response to influenza infection. (**A–E**) BALB/c mice were infected with PR8 (0.1LD_50_) i.n. (**A**) Absolute numbers of neutrophils (solid line) and inflammatory mononuclear cells (dashed line) infiltrating the respiratory tract over time. (**B**) Absolute numbers of lymphocytes (T cells – gray dashes; B cells – black dashes; NK cells – solid lines) infiltrating the respiratory tract over time. (**C**) The number of lung antigen-specific CD8^+^ T cells was estimated following *in vitro* re-stimulation with influenza-infected target cells (P815) and analysis of IFNγ^+^CD8^+^ T cells via flow cytometry. (**D**) IFNγ content and (**E**) viral titer in sampled BAL fluid over time. Dashed line signifies limit of viral detection. (**A–E**) Two-three independent experiments (n = 2–4/expt) represented as mean ± SEM.

Inflammatory cells representative of both the innate and adaptive immune system accumulate over time in the infected lung interstitium in response to IAV infection. Kinetic analysis of the accumulation of mononuclear and granulocytic innate immune cells in the lungs following sub-lethal infection of mice with the mouse-adapted IAV strain, A/Puerto Rico/8/34 (PR8) revealed that inflammatory mononuclear cells of the dendritic cell (DC)/macrophage lineage (so called exudate macrophages, TNF iNOS-producing DCs [15], [16]; CD45^+^, Ly6C^hi^, Ly6G^−^, CD11b^hi^, CD11c^hi^) were the most prominent innate immune cell type identified with maximum accumulation occurring approximately 10–12 days post infection (dpi) (Figure 1A and see Figure S1 for gating strategy). Neutrophils (CD45^+^, Ly6C^int^, Ly6G^hi^, CD11b^hi^) were likewise prominently represented in the inflammatory infiltrate (Figure 1A and Figure S1). As expected, neutrophils were first detected prior to the influx of inflammatory mononuclear cells (i.e. 2 dpi), peaked at 5–6 dpi, and decreased in number gradually following the resolution of infection (Figure 1A).

T lymphocytes (FSC^lo^, SSC^lo^, CD45^+^, Thy^+^, TCRβ^+^) were the dominant lymphocytic cell subset infiltrating the lungs during influenza infection. T lymphocytes reached similar numbers and exhibited similar kinetics of peak accumulation (i.e. 10–12 dpi) (Figure 1B) as inflammatory mononuclear cells (Figure 1A). Influenza antigen (ag)-specific effector CD8^+^ T cells accumulated with a similar kinetics as total T lymphocytes with the notable exception that the ag-specific CD8^+^ T cells were first detected between 4–6 dpi, that is, following their generation in and subsequent egress from the draining lymph nodes (DLNs) (Figure 1C). This early influx of ag-specific CD8^+^ T cells into the lung interstitium was associated with the release of the signature effector T cell cytokine, IFNγ, into the infected lungs as detected in bronchoalveolar lavage (BAL) fluid (Figure 1D). The early influx of effector T cells from the DLN into the infected lungs and the production of effector cytokines likewise paralleled the kinetics of virus elimination from the infected lungs (Figure 1E). Two other lymphocytic cell subsets, B cells (FSC^lo^,SSC^lo^, CD45^+^, B220^+^, TCRβ^−^) and NK cells (FSC^lo^, SSC^lo^, CD45^+^, B220^−^, TCRβ^−^; Dx5^+^), accumulated in substantial numbers in the infected lung interstitium with the tempo comparable to that of infiltrating T lymphocytes (Figure 1B).

### Influenza antigen expression by lung infiltrating CD45^+^ cells

Although virus clearance from the infected respiratory tract and specific elimination of infected respiratory epithelial cells is critical for control of influenza infection and recovery [4], [5], [6], we recently reported that effector cytokine production by anti-viral CD8^+^ T cells in the influenza-infected lungs is triggered not by the infected respiratory epithelium but rather by CD45^+^ inflammatory cells infiltrating the infected lungs [5]. It was therefore of interest to assess the kinetics and level of expression of influenza nucleocapsid protein (NP) by these lung infiltrating inflammatory cells since NP is the most abundant prototypical influenza antigen within the cytoplasm and nucleus of the IAV infected cell and is a major target of ag-specific effector CD8^+^ T cells. We prepared lung cell suspensions from infected lungs at various dpi. We fixed and permeabilized cells following cell surface marker staining and probed the permeabilized cells for the expression of intracellular NP using a flow cytometry based analysis.

**Figure 2:**
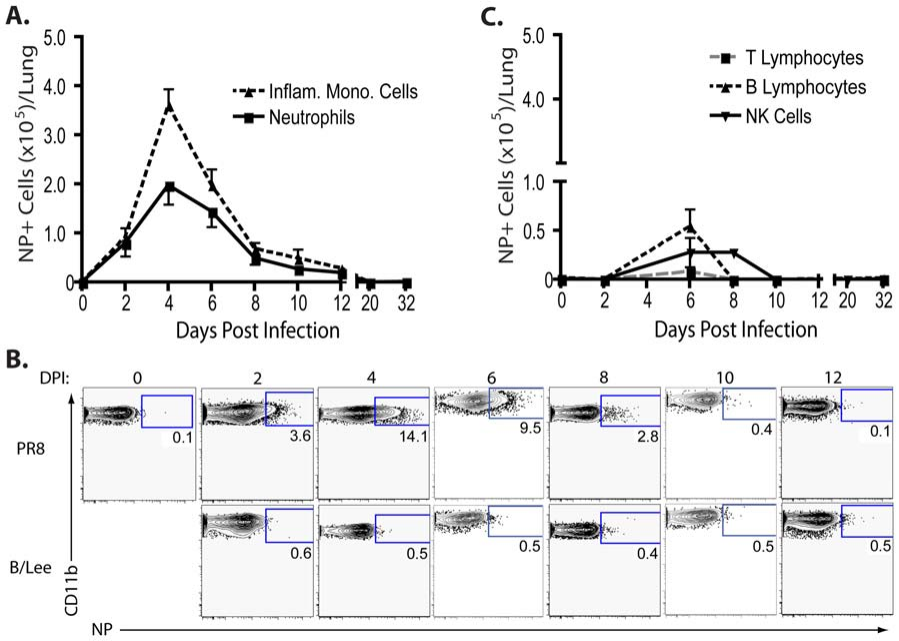
Hematopoietic cell influenza nucleocapsid protein content during an influenza virus infection. (**A–C**) BALB/c mice were infected with PR8 (0.1LD_50_) i.n.. (**A**) Absolute numbers of NP^+^ neutrophils (solid line) and NP^+^ inflammatory mononuclear cells (dashed line) infiltrating the respiratory tract over time. (**B**) Representative flow cytometry panels depicting influenza NP expression in the lung neutrophil population during the course of an influenza virus infection. Neutrophils from PR8 (top panels) or B/Lee (bottom panels) infected mice are depicted. (**C**) Absolute numbers of NP^+^ lymphocytes (T cells – gray dashes; B cells – black dashes; NK cells – solid lines) infiltrating the respiratory tract over time. (**A–C**) Cells from B/Lee infected mice (at corresponding day p.i.) were utilized to set flow cytometric gate due to the inability of the αNP to recognize B/Lee NP. Two independent experiments (n = 2–4/expt) represented as mean ± SEM where applicable.

As Figure 2A demonstrates, the inflammatory mononuclear cells were the dominant NP bearing cell population in the infected lungs, with maximum accumulation of NP^+^ inflammatory mononuclear cells (∼10–30% of lung infiltrating mononuclear cells) at peak virus replication (i.e. 4 dpi) (Figure 1E). Furthermore, the number of NP^+^ inflammatory mononuclear cells decreased (Figure 2A) as infectious virus was eliminated from the infected lungs (Figure 1E). We also observed a significant fraction (∼5–15%) of lung infiltrating granulocytic cells with the phenotypic characteristics of neutrophils (i.e. Ly6G^hi^, CD11b^hi^) expressing NP protein (Figure 2A). NP^+^ granulocytes were enumerated over time using granulocytes from the lungs of mice infected with type B influenza strain B/Lee as the gating control (Figure 2B) The tempo of NP expression by neutrophils was comparable to that of infiltrating inflammatory mononuclear cells and followed the kinetics of influenza replication and clearance in the infected lungs (Figure 1E). The expression of NP was not a feature of all inflammatory cells infiltrating the infected lungs. Only a small fraction of lung infiltrating B lymphocytes (∼1–2%) were NP positive and even smaller numbers of NK and T cells expressed this antigen (<1–2%) (Figure 2C), though the latter cell type was the major lymphocyte population infiltrating the infected lungs (Figure 1B).

### Neutrophils are infected by influenza virus

**Figure 3:**
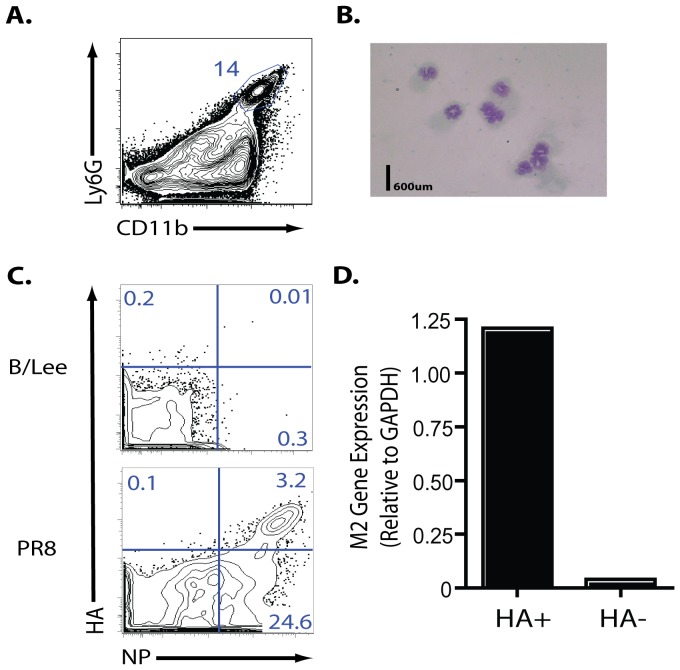
De *novo* influenza mRNA expression in lung neutrophils. (**A–D**) BALB/c mice were infected with PR8 (10LD_50_) i.n.. Cells were collected from 4 dpi animals (**A**) BAL fluid flow profile of percent of neutrophils (Ly6G^+^CD11b^hi^). Cells gated previously as CD45^+^Thy1.2^−^. (**B**) DiffQuick staining of sorted neutrophils (CD45^+^Ly6G^+^ Ly6C^int^CD11b^hi^) (**C**) Representative flow profile of HA^+^NP^+^ neutrophils at 4 dpi (Cells gated as CD45^+^Thy1.2^−^Ly6G^+^CD11b^hi^). B/Lee infected mice were utilized to set flow cytometric gate. (**D**) Influenza M2 mRNA expression (relative to GAPDH) from sorted HA^+^ and HA^−^ neutrophils (CD45^+^Ly6G^+^ Ly6C^int^CD11b^hi^). Table is representative of one experiment. Additional experiment yielded similar results. (**A–D**) Two independent experiments (n = 2–4/expt) represented as mean ± SEM (where applicable).

Unlike most RNA viruses, the replication of the influenza genome occurs within the nucleus of the virally-infected cell [17]. Since mature circulating neutrophils are short lived and display condensed chromatin, features typical of cells with minimal gene expression [18], it seemed most likely that neutrophils infiltrating the infected lungs would display influenza NP because these cells had taken up NP present in virus or dead/dying infected cells in the lungs. To further establish that the cells identified were neutrophils, we sorted the CD45^+^, Ly6C^int^, Ly6G^hi^, CD11b^hi^ cell subset (Figure 3A) and examined the cells for granulocytic morphology. As Figure 3B demonstrates, the Ly6G^hi^, CD11b^hi^ cell subset had the characteristic morphology of neutrophils.

Since, as noted above, uptake of NP from dead/dying infected cells or influenza virions was the most likely explanation for NP expression by lung infiltrating neutrophils, it was of interest to determine if viral antigen expression was due to the ability of the cells to be infected by IAV. We reasoned that phagocytic uptake of infected dying cells or virus would localize influenza proteins such as NP and presumably type A virus hemagglutinin (HA) to an intracellular endosomal compartment. By contrast following infection, the influenza NP would remain intracellular (i.e. requiring cell permeabilization to detect the cytoplasmic/nuclear NP by flow cytometry), but the newly synthesized HA glycoprotein would be detectable on the cell surface prior to fixation and permeabilization. We carried out this flow-based analysis on neutrophils isolated at the peak of PR8 infection and found that ∼10% of NP^+^ neutrophils simultaneously expressed the viral HA on their surface (Figure 3C). Importantly, while we could detect HA^−^NP^+^ neutrophils, we were unable to detect any cells expressing HA exclusively (HA^+^NP^−^). The specificity of this detection strategy was verified by the failure of these antibodies to detect type A influenza proteins in neutrophils taken from mice infected with the influenza B/Lee strain (Figure 3C).

To further establish that the lung infiltrating neutrophils were infected by IAV, we analyzed RNA from sorted HA^+^ and HA^−^ neutrophils for expression of the spliced mRNA encoding the influenza M2 gene production which is only present within infected cells. As Figure 3D demonstrates, we detected M2 gene expression primarily (but not exclusively) in the sorted HA^+^ lung neutrophils thereby establishing the capacity of mature tissue infiltrating neutrophils exposed to influenza to support *de novo* influenza virus gene expression.

**Figure 4:**
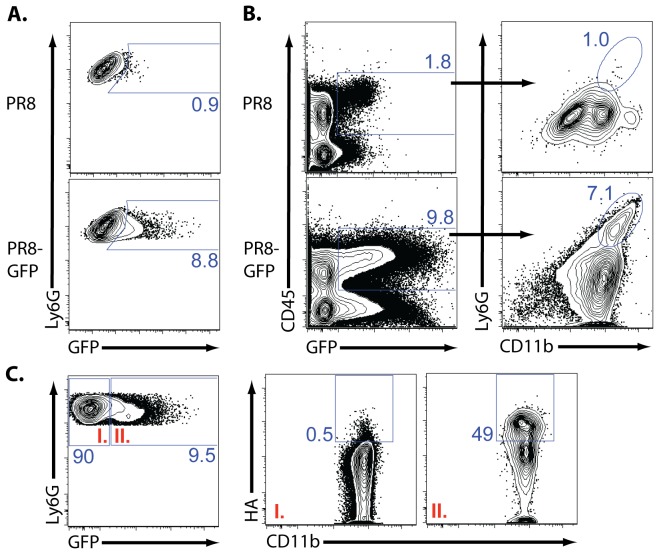
De *novo* influenza protein expression in lung neutrophils. (**A–B**) C57BL/6 mice were infected with NS-GFP or PR8 (10LD_50_) influenza virus. At 5 dpi, total lungs were collected. Representative flow profile of (**A**) total lung neutrophil GFP expression and (**B**) the percent of neutrophils present of total GFP^+^ hematopoietic cells. (**A–B**) Two independent experiments (n = 3–4/expt). (**C**) BALB/c mice were infected with NS-GFP influenza virus. At 5 dpi, total lung was collected. Representative flow profile GFP^+^ and GFP^−^ neutrophil HA staining. Two independent experiments (n = 2/expt). (**A–C**) Neutrophils gated as CD45^+^Ly6G^+^CD11b^hi^. GFP gate was set utilizing splenic cells from infected animals. HA gate was set utilizing isotype control.

To complement and support these findings, we next utilized a reverse genetics engineered PR8 strain which expresses the GFP gene within the influenza non-structural (NS) gene, in such a way as to not disrupt NS gene expression [3]. Because the NS-encoded GFP protein is not incorporated into the virion itself but is only expressed within virally-infected cells, we could use GFP protein expression during NS-GFP virus infection *in vivo* to identify infected cells (i.e cells undergoing *de novo* influenza protein synthesis). Following NS-GFP virus infection of C57BL/6 mice, we detected GFP expression in approximately 10% of the neutrophil subset (Ly6G^hi^, CD11b^hi^) (Figure 4A). Similar results were seen in BALB/c mice (Figure 4C). Hematopoietic cells from mice infected with the wt influenza virus demonstrated a modest level of fluorescence in the GFP channel (most likely representing background fluorescence), but this was not associated with the neutrophil subset (Figure 4B). As expected, surface expression of HA on neutrophils was associated only with GFP^+^ cells (Figure 4C) while a somewhat larger fraction of GFP^−^ cells (i.e. 20%) expressed intracellular influenza NP antigen (data not shown).

### Neutrophils serve as APC for influenza-specific effector CD8^+^ T cells

**Figure 5:**
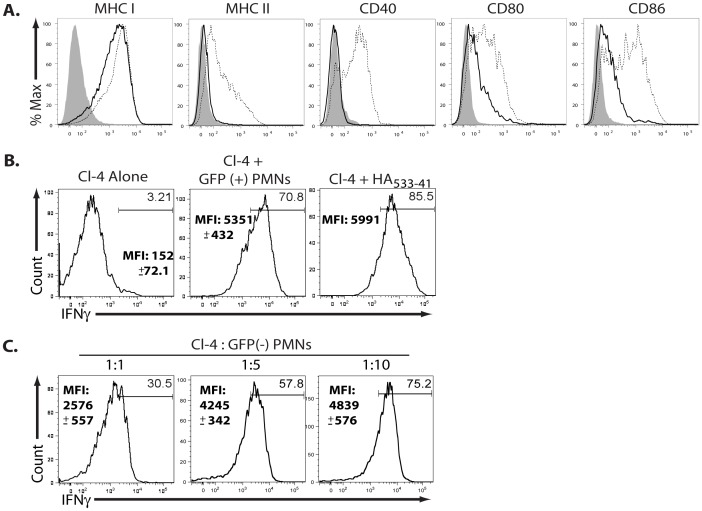
Neutrophils can stimulate CD8^+^ T cell pro-inflammatory cytokine production *in vitro*. (**A**) BALB/c mice were infected with PR8 (0.1LD_50_) and lungs were collected at 6 dpi. Inflammatory mononuclear cells (dashed lines) and neutrophil (solid line) MHC and co-stimulatory molecule expression is depicted. Isotype control is shaded area. Two independent experiments (n = 2–4/expt). (**B–C**) BALB/c mice were infected with NS-GFP influenza virus. At 5 dpi, lung neutrophils (CD45^+^, Thy^−^, Ly6G^hi^, Ly6C^int^, CD11b^hi^) were sorted based on GFP expression. In parallel, 2×10^5^ CL-4 CD8^+^Thy1.1^+^ were adoptively transferred into Thy1.2^+^ BALB/c mice and were infected with PR8 (0.1LD_50_) the following day. At 8 dpi, lung CL-4 cells (CD45^+^, Thy1.2^−^, Thy1.1^+^CD4^−^,CD8^+^) were sorted and cultured (**B**) in a 1∶1 ratio with GFP^+^ neutrophils or (**C**) in depicted effector∶target ratios with GFP^−^ neutrophils. (**B–C**) Cells were co-cultured for six hours in the presence of GolgiStop. Representative flow profiles depict CL-4 T cell (CD45^+^CD8^+^ Thy1.1^+^ FSC^lo^SSC^lo^) IFNγ production. HA_533–41_ pulsed CL-4 T cells served as a positive control. Mean fluorescence intensity (MFI) is depicted with standard deviation. Two independent experiments (n = 2/expt).

The above results demonstrated that neutrophils could support *de novo* IAV gene expression *in vivo* within the infected lungs and that both the infected (HA^+^NP^+^GFP^+^) neutrophils as well as the presumably uninfected (HA^−^NP^+^GFP^−^) NP antigen containing neutrophils were present at the peak of CD8^+^ T cell effector activity. In view of these findings, we asked whether these lung-infiltrating neutrophil populations could act as APC for IAV-specific effector CD8^+^ T cells. We have previously demonstrated that the triggering of cytokine production by effector CD8^+^ T cells in the infected respiratory tract requires TCR engagement of MHC Class I/IAV antigen complexes as well as interaction with co-stimulatory ligands, notably CD80 and CD86 [5]. As depicted in Figure 5A, neutrophils isolated from the infected lung indeed expressed equivalent levels of MHC I as inflammatory mononuclear cells and express CD80 and CD86, at only slightly lower levels. In contrast, neutrophils did not express MHC Class II, which is necessary for CD4^+^ T cell recognition of antigen.

To evaluate the *in vitro* APC activity of neutrophils exposed to IAV *in vivo*, we purified by cell sorting GFP^+^ (infected) and GFP^−^ (uninfected) neutrophils from the lungs of mice infected with the NS-GFP virus. In order to ensure that the *in vitro* interaction of lung derived neutrophils with effector T cells as closely as possible mimicked the interaction *in vivo*, we used as a defined source of effector CD8^+^ T cells for *in vitro* co-culture with neutrophils, PR8 (HA_533–41_ epitope) specific CD8^+^ TCR transgenic (tg) clone 4 (Cl-4) T cells isolated from PR8 infected lungs 8 days after adoptive transfer and virus infection. Thus, both the neutrophils and the effector CD8^+^ T cells employed in this *in vitro* analysis were isolated from the infected lungs. Infected (GFP^+^) neutrophils had potent APC activity as reflected through IFNγ production by Cl-4 T cells in the *in vitro* intracellular cytokine staining (ICCS) assay, at T cell∶APC ratios as low as 1∶1 (Figure 5B). It is noteworthy that the frequency of IFNγ^+^ T cells stimulated by the infected neutrophils was comparable to that of un-infected neutrophils pulsed with the synthetic HA_533–41_ peptide epitope recognized by Cl-4 T cells. By contrast, un-infected (GFP^−^) neutrophils co-cultured with Cl-4 T cells at a comparable ratio exhibited weaker APC activity than infected cells (i.e. frequency and MFI of Cl-4 IFNγ production; Figure 5C). However, while not as potent APC as infected neutrophils, GFP^−^ neutrophils were capable of triggering the IAV HA-specific Cl-4 T cells when the cells were co cultured at higher T cell∶neutrophil ratios.

**Figure 6:**
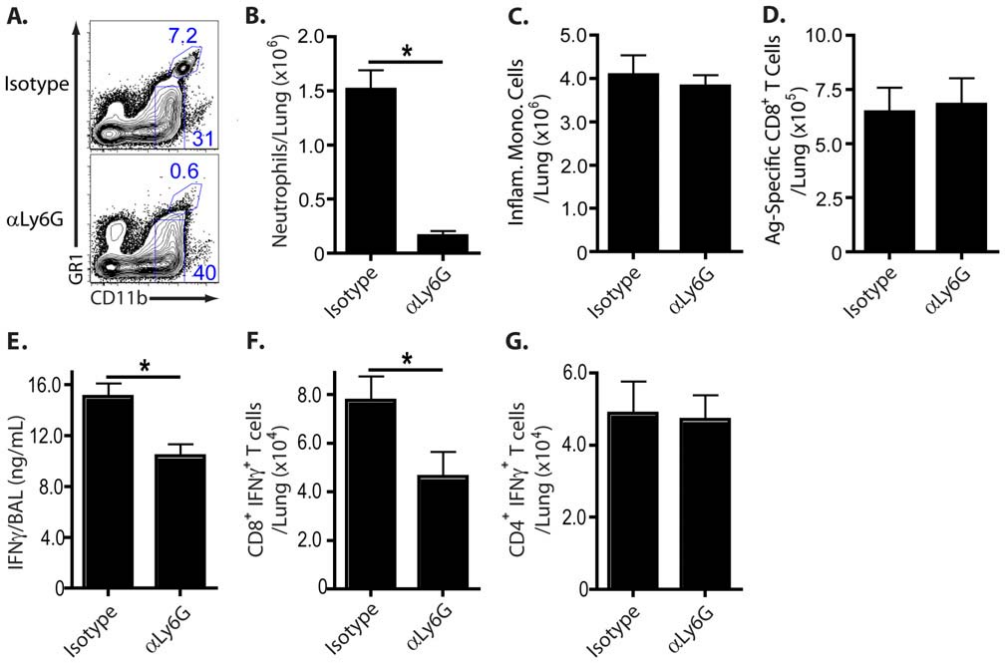
Neutrophils trigger CD8^+^, but not CD4^+^, T cell anti-viral activity *in vivo*. (**A–G**) BALB/c mice were infected with PR8 (0.1LD_50_) and at 5 dpi, were administered 500 ug isotype or αLy6G depleting antibody i.p.. Lung tissue/BAL fluid was collected twenty-four hours later. (**A**) Representative flow profile depicting neutrophil depletion (previously gated as CD45^+^Thy1.2^−^). Absolute number of lung (**B**) neutrophils, (**C**) inflammatory mononuclear cells, and (**D**) antigen-specific CD8^+^ T cells in isotype or depleting antibody treated mice. (**B**) Neutrophils were gated as CD45^+^, GR1^hi^, CD11b^hi^, FSC^lo^, SSC^int^ and (**C**) inflammatory mononuclear cells as CD45^+^, Ly6C^hi^, GR1^int/lo^, CD11b^hi^,MHC II^hi^. (**D**) Antigen-specific CD8^+^ T cells was estimated following *in vitro* re-stimulation with influenza-infected target cells (P815) and analysis of IFNγ^+^CD8^+^ T cells via flow cytometry. (**E**) IFNγ content in sample BAL fluid. (**F–G**) Six hours prior to harvest, monensin was administred i.p. to mice (*in vivo* ICCS assay). The number of (**F**) CD8^+^ or (**G**) CD4^+^ T cells producing IFNγ with and without neutrophil depletion is depicted. (**A–D**) Three independent experiments (n = 2–3/expt) represented as mean ± SEM (where applicable). Considered statically significant at P<.05 (*).

The above *in vitro* findings supported the possibility that like inflammatory mononuclear cells present in the lungs of influenza-infected mice [5], neutrophils may also serve as APC for lung infiltrating effector CD8^+^ T cells *in vivo*. To directly address this possibility we examined the impact of acute antibody mediated (αLy6G) *in vivo* depletion of neutrophils from infected mice at 5 dpi on effector (i.e. IFNγ) cytokine production by CD8^+^ T cells 24 hours later. Acute administration of the Ly6G depleting antibody reduced neutrophil numbers by 90% 24 hours later (i.e. 6 dpi), which is also the peak of CD8^+^ T cell cytokine production (Figure 6A and 6B). Importantly, acute neutrophil depletion at 5 dpi had no effect either on lung virus titer (data not shown), the frequency of inflammatory mononuclear cells, or the frequency of IAV specific effector CD8^+^ T cells in the infected lungs (as measured by the *in vitro* ICCS assay) (Figure 6C and 6D). Neutrophil depletion did result in a substantial decrease in IFNγ release into the bronchoalveolar lavage (BAL) fluid 24 hours later (i.e. 6 dpi) compared to isotype antibody-treated infected mice (Figure 6E). IL-10, the regulatory cytokine which is primarily produced by effector CD8^+^ T cells during IAV infection [19], was also diminished in neutrophil depleted mice compared to controls, though this was variable from experiment to experiment (data not shown).

Although our laboratory has previously reported that IFNγ released into the BAL fluid is principally T cell-derived during influenza infection [5], [19], we wanted to further ensure that the reduction in mediators release following acute neutrophils depletion was due to the impact of the elimination of this cell type on the function of CD8^+^ T cells *in vivo*. To identify T cells actively producing IFNγ *in vivo*, we employed a modified form of the *in vivo* ICCS assay [5], [20], where the protein transport inhibitor monensin is administered into infected mice to prevent T cell cytokine release. When T cells producing IFNγ *in vivo* were enumerated using this assay, we found, consistent with the cytokine release data from the BAL fluid, that there was a statistically significant decrease in the numbers of CD8^+^ T cells secreting IFNγ (IFNγ^+^CD8^+^ T cells) *in vivo* compared to the corresponding isotype controls (Figure 6F). Neutrophil depletion only affected CD8^+^ T cell IFNγ production *in vivo* as the absence of neutrophils had no apparent impact on the migration, accumulation, or frequency of ag-specific CD8^+^ T cells into/within the infected respiratory tract (Figure 6D). Importantly, the acute neutrophils depletion had no effect on IFNγ producing CD4^+^ T cells (Figure 6G) consistent with the fact that neutrophils express no MHC Class II and would not be expected to serve as APCs for anti-viral effector CD4^+^ T cells *in vivo* (Figure 5A). Thus, while acute neutrophil depletion has no effect on the frequency of antigen specific CD8^+^ T cells trafficking to the infected lungs during the 24 hours following depletion, the elimination of neutrophil APC activity did diminish the efficiency of mediator release by the effector CD8^+^ T cells

## Discussion

In this study, we characterized the response of neutrophils infiltrating the lungs following IAV infection. We observed that neutrophils and inflammatory mononuclear cells were the most abundant myeloid lineage cells present in the infected lungs at the peak of CD8^+^ T cell effector activity (i.e. day 6 p.i.) when cytokine production by effector CD8^+^ T cell was maximal. Although the lymphoid lineage cells were also present in large numbers in the IAV infected lungs at this time, only neutrophils and inflammatory mononuclear cells contained intracellular deposits of the IAV nucleocapsid protein (i.e. NP^+^ cells) in significant numbers. Among the NP^+^ neutrophils, up to 10% were shown to be infected as they simultaneously expressed both intracellular NP and cell surface HA. This is probably an underestimate of the true frequency of infected neutrophils if the tempo of NP and HA protein expression in the infected neutrophils differ (i.e. NP expression early post infection and HA expression later). In support of the infected status of the NP^+^HA^+^ lung neutrophils, the cells were substantially enriched for expression of the spliced M2 mRNA compared to NP^+^HA^−^ neutrophils. We further confirmed that neutrophils infiltrating the IAV infected lungs were susceptible to infection (*de novo* viral gene expression) using the GFP reporter recombinant IAV strain. Employing GFP expression as an indicator of IAV infection of neutrophils, we separated GFP^+^ and GFP^−^ neutrophils from the infected lungs and demonstrated that GFP^+^ neutrophils were potent *in vitro* stimulators of cytokine production by effector CD8^+^ T cells isolated from the IAV infected lungs. We further demonstrated that acute elimination of neutrophils *in vivo* significantly reduced total effector cytokine release in the infected lungs and diminished the frequency of IFNγ secreting effector CD8^+^ T cells *in vivo* without altering effector CD4^+^ T cell responses to IAV in the infected lungs.

The finding that a small but nevertheless substantial fraction of the neutrophils infiltrating the infected lungs are susceptible to influenza infection was not anticipated. Mature neutrophils are short lived within the respiratory tract [21], contain a high ratio of heterochromatin to euchromatin [18], [22], and are generally considered to be largely transcriptionally inactive [21]. These properties of neutrophils suggest a cellular environment which would be non-permissive for an RNA virus like IAV, whose genome replicates in the nucleus and is dependent on nuclear localized host factors [12], [17]. In support of this concept, bone marrow (BM)-derived neutrophils were recently shown to be resistant to influenza infection *in vitro* [23]. However, during phagocytosis, neutrophils do undergo active transcription and can be demonstrated to up-regulate transcriptional activity in response to bacterial and viral infections [21], which may allow this cell type to support infection (i.e. *de novo* gene expression, following infection by certain organisms). Indeed, neutrophils have been shown to support infection by intracellular bacteria [21], herpes viruses [24], [25], and most recently, the engineered NS-GFP reporter IAV strain [3]. The failure to detect infection of neutrophils by IAV in previous reports may be due to underappreciated differences in the activation state (e.g. transcriptional activity) between neutrophils isolated from the bone marrow or circulation with subsequent infection *in vitro* [12], [23] and tissue infiltrating neutrophils exposed to IAV *in situ* [3]. In this connection, it is important to note the likelihood of species dependent differences in susceptibility of neutrophils to infection since circulating human neutrophils may be susceptible to infection by IAV (i.e. able to take up the virus when exposed to IAV *ex vivo* and allow expression of IAV genes in short-term culture) [26], [27], [28]. Furthermore in at least one report of lethal human infection with highly pathogenic avian H5N1 IAV, neutrophils were shown to contain IAV protein and RNA. However whether this was due to direct infection or uptake of virus/infected cellular material was not determined [28].

The expression of the virus GFP reporter gene in neutrophils, the detection of cell surface HA, and the presence of elevated levels of spliced viral M2 mRNA in the HA^+^GFP^+^ neutrophils represent rigorous criteria that neutrophils are infected by IAV *in vivo*. Infected neutrophils serve as potent APC for effector CD8^+^ T cells in short term *in vitro* culture. The likely explanation for their potency is that direct infection provides the most efficient mechanism to generate and load processed viral peptides onto MHC I molecules. Although we detect minimal differences in the expression of MHC I molecules and conventional accessory molecules (e.g. co-stimulatory ligands CD80/86) between GFP^+^ and GFP^−^ neutrophils (data not shown), we cannot formally exclude the possibility of an infection induced alteration in expression of cell surface or soluble molecules in the infected cells which accounts for efficient triggering of effector CD8^+^ T cells. We also observed that only approximately 50% of infected/GFP^+^ neutrophils were simultaneously HA^+/hi^. While this could reflect a failure to express the full complement of IAV genes in this cell type, a more likely explanation, as noted above, is that viral gene expression is not coordinated (i.e. NS gene expression proceeds at a faster rate than HA).

Uninfected (GFP^−^HA^−^) neutrophils included both NP^+^ and NP^−^ cells. This cell population could also serve as APC for effector CD8^+^ T cells *in vitro* suggesting that within this neutrophil population were cells displaying the processed HA epitope recognized by the TCR tg CL-4 T cells. The likeliest explanation for the capacity of these cells to stimulate effector CD8^+^ T cells is that the neutrophils in the infected lungs have taken up IAV antigens (e.g. HA) derived from virions and/or infected cell remnants and cross presented processed viral antigen to the T cells. To our knowledge, this is the first demonstration in the murine system (following natural infection) of processing and cross presentation of viral antigens to CD8 T cells although murine neutrophils have previously been reported to cross present soluble antigen *in vivo* and bacterial antigen *in vitro* to CD8^+^ T cells [29], [30]. We cannot formally evaluate if antigen-presenting activity is restricted to the NP^+^ subset of uninfected neutrophils since detection of the intracellular NP requires permeabilization of the cells (with loss of viability) prior to cell sorting. If only a fraction of the uninfected neutrophils (i.e. the NP^+^ cells) display the relevant peptide MHC complex, this could in part account for the lower efficiency of effector CD8 T cells stimulation by the uninfected (GFP^−^) neutrophil subset.

We and others have previously identified CD11c^hi^ inflammatory mononuclear cells as stimulators of CD8^+^ T cell effector activity *in vivo* [5], [31], [32]. Our findings both *in vitro* and *in vivo* implicate neutrophils as a second inflammatory cell type capable of acting as a potent APC for effector CD8^+^ T cells during the adaptive immune response to IAV in the lungs. Our observation that acute depletion of neutrophils *in vivo* significantly diminished the release of IFNγ by effector CD8^+^ T cells in the lungs strongly supports this view. We had also noted in the infected lungs of mice depleted of neutrophils an apparent, but not statistically significant, reduction in the release into the BAL fluid of the regulatory cytokine IL-10 (data not shown), which we have previously identified as a major CD8^+^ T cell product during IAV infection [19], [33]. The significance of this difference in the impact of neutrophil depletion on IFNγ and IL-10 production by effector CD8^+^ T cells in the lungs is currently unclear and will require further investigation. Other T cell derived cytokines were modestly affected by acute neutrophil depletion, as well (e.g. MIP-1α; data not shown); however, many infiltrating immune cell types as well as resident lung cells produce these cytokines complicating the interpretation of the results. It is formally possible that neutrophils function indirectly to support or enhance the soluble mediator response of CD8 T cells by recruiting, activating, and/or serving as a IAV antigen reservoir for MHC class I and II^+^ CD11c^hi^ inflammatory mononuclear cells [30], [34], [35], [36], [37]. Because of the minimal impact of the acute neutrophil depletion *in vivo* on CD4^+^ T cell derived IFNγ production, we favor a direct role of neutrophils as APC for CD8^+^ T cells. Thus, we conclude neutrophils, as well as CD11c^hi^ inflammatory mononuclear cells, are key APCs regulating the pro-inflammatory and regulatory cytokine production by effector CD8^+^ T cells in the IAV-infected lung *in vivo*.

In conclusion in this report, we demonstrate that neutrophils are capable of both being infected by IAV and acquiring viral antigen by phagocytosis of infectious virus or infected cells. Neutrophils and in particular infected neutrophils act as potent APC to trigger the release of soluble mediators by effector CD8^+^ T cells responding in the infected lungs to IAV infection. It remains to be determined whether there is a virus strain dependent difference in the susceptibility of neutrophils infiltrating the infected lungs to IAV infection and whether infection of neutrophils by IAV in lungs impacts on the pathogenesis of IAV infection and the innate and adaptive immune response to infection.

## Materials and Methods

### Ethics Statement

This study was carried out in strict accordance with the Animal Welfare Act (Public Law 91-579) and the recommendations in the Guide for the Care and Use of Laboratory Animals of the National Institutes of Health (OLAW/NIH, 2002). All animal experiments were performed in accordance with protocols approved by the University of Virginia Animal Care and Use Committee (ACUC; Protocol Number 2230).

### Mice and infection

Female BALB/c, C57BL/6, and Thy-1.1 CL-4 tg mice (BALB/c background) were purchased from the National Cancer Institute and Jackson Laboratories. All mice were housed in a pathogen-free environment and used at 8–14 weeks of age for all experiments. NS-GFP virus was a generous gift from the Adolfo Garcia-Sastre laboratory [3]. Type A influenza viruses PR8 (H1N1), NS-GFP (H1N1), and type B influenza B/Lee were grown in day 10 chicken embryo allantoic cavities as described previously [38]. Mice were infected with 250 EID PR8 (0.1LD_50_), 25,000 EID PR8 (10LD_50_), 10^5^ EID NS-GFP, or 10^5^ EID B/Lee. All infectious doses were administered i.n. in 50 µL (diluted in serum-free Iscove's medium (Invitrogen)) following ketamine and xylazine anesthesia.

### Tissue preparation

Mice were euthanized via cervical dislocation. Lungs were perfused via the right ventricle of the heart with PBS and were enzymatically digested with Type II collagenase (37°C for 30 minutes; Worthington), followed by passing through a steel screen. Red blood cells in the cell suspensions were lysed using ammonium chloride. Cells were counted using a hemacytometer after Trypan blue dye exclusion and resuspended in FACS buffer containing PBS, 2% FBS, 10 mM EDTA, and 0.01% sodium azide for Ab staining or MACS buffer containing PBS, 2% FBS, and 10 mM EDTA for sorting.

### Antibodies

The following monoclonal antibodies (mAb) were purchased from BD-Biosciences (BD; San Diego, CA) or eBiosciences (eBio; San Diego, CA)(unless stated), as conjugated to FITC, Alexa-488, PE, PE-Cy7, PerCP-Cy5.5, APC, Alexa-647, APC-Alexa780 or Biotin: CD4 (GK1.5), CD8α (53-6.7), CD11b (M1/70), CD11c (HL3), CD45 (30-F11), CD80 (16-10A1), CD86 (GL-1), CD90.1 (OX-7), CD90.2 (53-2.1), Gr-1 (RB6-8C5), Ly6G (1A8), Ly6C (AL-21), H-2K^b^ (AF6-88.5), H-2K^d^ (SF1-1.1), I-A^d^ (AMS-32-1), IFNγ (XMG1.2), and HA (Kind gift from Dr. Jon Yewdell, NIH/NIAID), isotype control antibodies. Anti-mouse CD16/32 and influenza NP (HB65) was isolated and purified in our laboratory. For biotinylated mABs, samples were incubated with streptavidin-PE.

### *In vitro* CD8^+^ T cell re-stimulation assay

To measure IFNγ stimulation, CD8^+^ T cells were co-cultured cells for 6 hours at 37°C in DMEM+5% FCS in the presence of Golgi-Stop (BD Biosciences, 1.6 µL mL^−1^). For identification of antigen-specific polyclonal CD8^+^ T cells, single-cell suspensions from BALB/c mice were co-cultured with infectious virus-pulsed P815 cells (10 MOI) in a 1∶1 ratio. The percent of CD8^+^ T cells stimulated to produce IFNγ as determined by flow cytometry was utilized to calculate total number of antigen-specific CD8^+^ T cells.

### Flow cytometry staining, analysis, and sorting

Cells suspensions were blocked with anti-mouse CD16/32 and then incubated with specific mAbs or isotype/FMO controls for 30 min at 4°C. Surface maker staining and intracellular cytokine staining were described previously [39]. Flow Cytometry was performed on FACS Canto flow cytometers (BD), and data were analyzed using FlowJo (Tree Star, Inc.). Neutrophils and T cells were sorted following MACS enrichment (Thy1.2 negative selection for neutrophils, CD8 positive selection for T cells) using a FACS Vantage SE Turbo sorter at the Flow Cytometry Core Facility (University of Virginia). Purity of neutrophils was confirmed by DiffQuick staining (IMEM Inc.) as per manufacturers' instructions.

### Flow cytometric detection of influenza-infected cells types

Cells suspensions were blocked with anti-mouse CD16/32 and then incubated with specific surface mAbs and HA antibody conjugated to biotin (Kind gift from Dr. Jon Yewdell, NIH/NIAID) for 30 min at 4°C. Cells were washed and then incubated with streptavidin-PE for ten minutes at 4°C. Cells were washed and were resuspended in Cytofix/Cytoperm solution (BD) according to the manufacturer's protocol. Cells were washed with PermWash (BD), and intracellular stained with NP antibody conjugated to Alexa-647 for thirty minutes at 4°C. Cell suspensions from B/Lee infected mice at equivalent days post infection were utilized to set flow cytometry gates.

### Bronchoalveolar lavage fluid (cytokine and viral titer)

We obtained BAL fluid by flushing the airways three times with a single use of 1 mL sterile PBS via a trachea incision. BAL fluid cytokine content was determined via ELISA (BD Biosciences) according to the manufacturer manual. Viral titer was determined via endpoint dilution assay and expressed as tissue culture infectious dose 50 (TCID_50_) units. We incubated Madin-Darby canine kidney cells (ATCC collection) with tenfold dilutions of BAL fluid from influenza virus-infected mice in serum-free DMEM culture+trypsin. After 3–4 day incubation at 37°C in a humidified atmosphere of 5% CO_2_, supernatants were collected and mixed with a half-volume of 1% chicken red blood cells (University of Virginia Veterinary Facilities). Hemagglutinin patterns were read thereafter and calculated as TCID_50_ values.

### Real-Time PCR

RNA from sorted cells was isolated as previously described [19]. We performed real-time RT-PCR in a StepOnePlue PCR System (Applied Biosystems) with SYBR Green PCR Master Mix (Applied Biosystems). Data were generated by the comparative threshold cycle 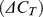 method by normalizing to GAPDH. The sequences of GAPDH primers used in the studies are available on request. Forward and reverse primers amplifying M2 vmRNA are as follows, respectively: 5′ – GAGGTCGAAACG CCT – 3′ and 5′ – CTGTTCCTTTCGATATTCTTCCC – 3′

### Neutrophil depletion

At noted day after infection with influenza, we injected mice with 500 µg Ly6G-specific mAb (clone IA8, BioExpress) or isotype, i.p..

### *In vivo* intracellular cytokine staining assay

Cytokine-producing cells *in vivo* were measured utilizing monensin on a previously described protocol [5].

### Statistics

An un-paired two-tailed T test was used to compare between treatment groups. Statistical analyses were performed using Prism3 software (for Macintosh; GraphPad Software, Inc.). Results are expressed as means ± SEM unless noted otherwise. Values of P<0.05 were considered statistically significant.

## Supporting Information

Figure S1 **Identification of neutrophils and inflammatory mononuclear cells in the infected lung.** Representative flow cytometry panels of the lung suspension collected from a day four post infected BALB/c mouse (A/PR/8/34; LD50 = 0.1). Neutrophils (A) were identified as CD45^+^Ly6G^+^CD11b^hi^Ly6C^int^. The cell type has the characteristic FSC/SSC profile of neutrophils. Inflammatory mononuclear cells (B) are a heterogenous immune infiltrate identified as CD45^+^Ly6G^−^CD11b^hi^Ly6C^hi^CD11c^hi^.
(TIF)
