# Supplementary figures and images for: Influenza-Infected Neutrophils within the Infected Lungs Act as Antigen Presenting Cells for Anti-Viral CD8+ T Cells
Source: PLoS One. 2012 Oct 8;7(10):e46581. doi: 10.1371/journal.pone.0046581 (PMC3466305; doi:10.1371/journal.pone.0046581)

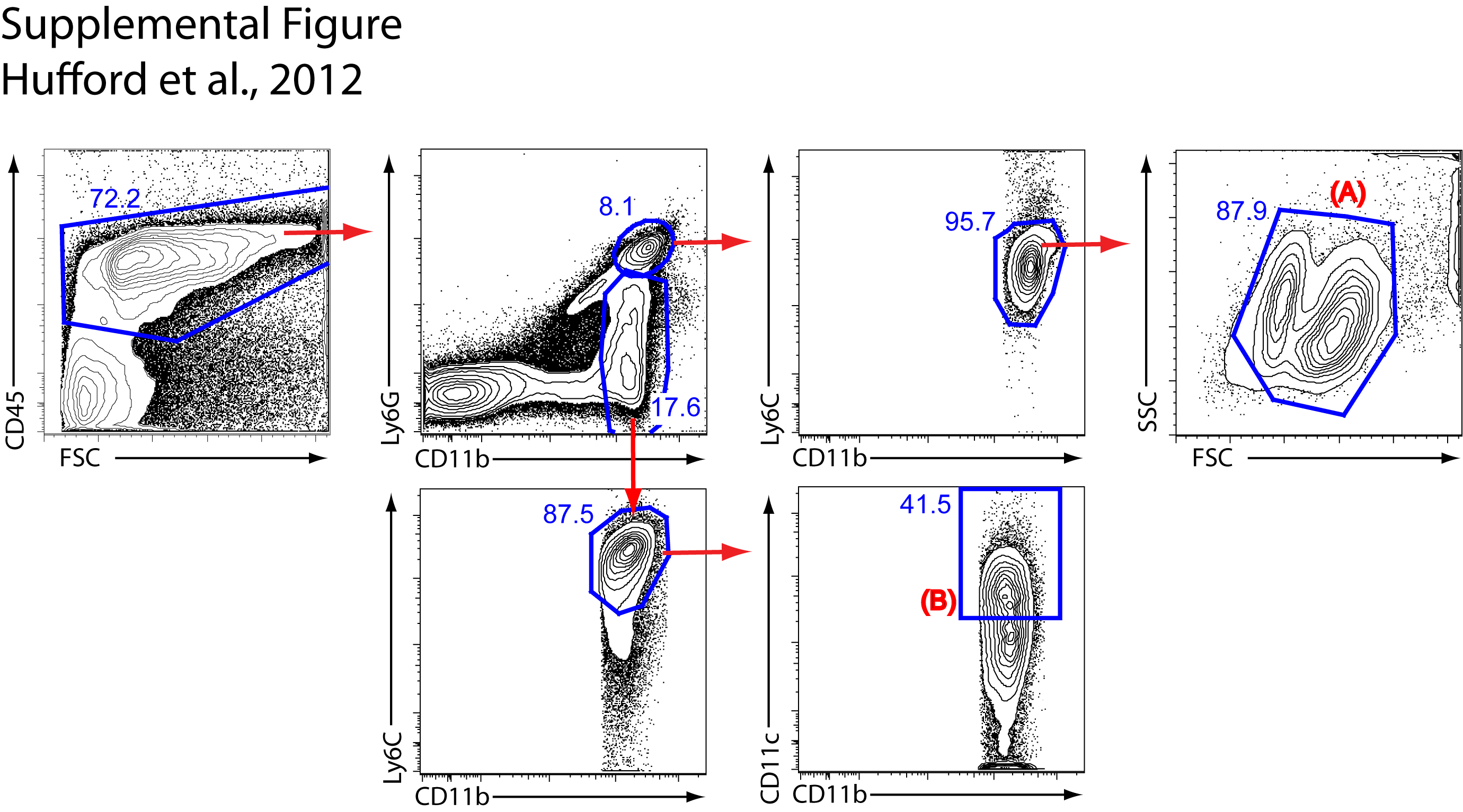

Supplement: Figure S1 — Identification of neutrophils and inflammatory mononuclear cells in the infected lung. Representative flow cytometry panels of the lung suspension collected from a day four post infected BALB/c mouse (A/PR/8/34; LD50 = 0.1). Neutrophils (A) were identified as CD45+Ly6G+CD11bhiLy6Cint. The cell type has the characteristic FSC/SSC profile of neutrophils. Inflammatory mononuclear cells (B) are a heterogenous immune infiltrate identified as CD45+Ly6G−CD11bhiLy6ChiCD11chi. (TIF) [file pone.0046581.s001.tif]
